# Evaluation of dosimetric uncertainty caused by MR geometric distortion in MRI‐based liver SBRT treatment planning

**DOI:** 10.1002/acm2.12520

**Published:** 2019-01-29

**Authors:** Silu Han, Fang‐Fang Yin, Jing Cai

**Affiliations:** ^1^ Medical Physics Graduate Program Duke University Medical Center Durham NC USA; ^2^ Department of Biomedical Engineering University of Arizona Tucson AZ USA; ^3^ Department of Radiation Oncology Duke University Medical Center Durham NC USA; ^4^ Department of Health Technology and Informatics The Hong Kong Polytechnic University Kowloon Hong Kong

**Keywords:** image distortion, liver cancer, MRI‐based treatment planning, radiation therapy, SBRT

## Abstract

**Purpose:**

MRI‐based treatment planning is a promising technique for liver stereotactic‐body radiation therapy (SBRT) treatment planning to improve target volume delineation and reduce radiation dose to normal tissues. MR geometric distortion, however, is a source of potential error in MRI‐based treatment planning. The aim of this study is to investigate dosimetric uncertainties caused by MRI geometric distortion in MRI‐based treatment planning for liver SBRT.

**Materials and Methods:**

The study was conducted using computer simulations. 3D MR geometric distortion was simulated using measured data in the literature. Planning MR images with distortions were generated by integrating the simulated 3D MR geometric distortion onto planning CT images. MRI‐based treatment plans were then generated on the planning MR images with two dose calculation methods: (1) using original CT numbers; and (2) using organ‐specific assigned CT numbers. Dosimetric uncertainties of various dose‐volume‐histogram parameters were determined as their differences between the simulated MRI‐based plans and the original clinical CT‐based plans for five liver SBRT cases.

**Results:**

The average simulated distortion for the five liver SBRT cases was 2.77 mm. In the case of using original CT numbers for dose calculation, the average dose uncertainties for target volumes and critical structures were <0.5 Gy, and the average target volume percentage at prescription dose uncertainties was 0.97%. In the case of using assigned CT numbers, the average dose uncertainties for target volumes and critical structures were <1.0 Gy, and the average target volume percentage at prescription dose uncertainties was 2.02%.

**Conclusions:**

Dosimetric uncertainties caused by MR geometric distortion in MRI‐based liver SBRT treatment planning was generally small (<1 Gy) when the distortion is 3 mm.

## 1 INTRODUCTION

The American Cancer Society estimates that in 2018, about 42 220 adults (30 610 in men and 11 610 in women) in the United States will be diagnosed with primary liver cancer.^1^ Liver is also a common site of metastases.^2^, ^3^ Nearly 70–90% of liver metastases cannot be resected through surgery.^3^ Stereotactic body radiotherapy (SBRT) has been shown to improve the local control rate of liver cancer. Different from the conventional radiation therapy which uses low fractional dose of ~2 Gy/fx, SBRT has a substantially greater cell‐killing effect using very high fractional dose of 10–20 Gy/fx,^4^, ^5^ leading to the excellent local tumor control rates of >90% if adequate radiation dose is delivered.^6^ Sharp dose fall off outside the target volume of SBRT requires precise contouring of target volume and organs at risk (OARs).^7^, ^8^ Advanced imaging techniques, such as 4D‐CT, are commonly used for precise tumor volume contouring in SBRT.

Current liver SBRT technique is CT‐based. However, CT is known to have low soft tissue contrast, and thus inability to accurately determine tumor volume and tumor motion in the abdomen.^9^, ^10^, ^11^, ^12^ In current liver SBRT treatment planning, MRI is often fused to CT to assist target volume delineation. This approach is not ideal as the registration between CT and MRI is prone to errors, and large safety margin is often needed to compensate for this uncertainty,^13^ which will increase the radiation dose to OARs. Therefore, current CT‐based liver SBRT treatment planning is ineffective and inefficient. It requires multiple imaging scans (CT, multiple MRI, etc.) and additional planning efforts (CT‐MRI registration, contour transfers, etc.), which increases the planning time, cost, and associated uncertainties. There is a clear need for improved liver SBRT technology.

Compared to CT, MRI has many significant advantages for radiotherapy planning, including superior tumor and soft‐tissue contrast, flexible imaging orientation, freedom from radiation exposure, and real‐time imaging. MRI‐based treatment planning is an emerging technique that can potentially improve tumor volume accuracy and dosimetry as compared to CT‐based planning for certain cases. MRI‐based treatment planning has been developed in brain, head and neck, and prostate. Paradis et al.^14^ proposed to generate synthetic CT images by segmenting brain tissues in MR images based on probabilistic classification using fuzzy c‐means clustering and assigning corresponding weighed CT number to each voxel. Dosimetric comparison was performed between CT‐based and MRI‐based brain volumetric‐modulated radiation therapy treatment planning. Hsu et al.^15^ investigated probabilistic tissue classification based on fuzzy c‐means clustering for soft tissue segmentation in head and neck, and performed CT number assignment according to the International Commission on Radiation Units and Measurements (ICRU) Report 46. Chen et al.^16^ illustrated MRI‐based treatment planning for prostate intensity‐modulated radiation therapy (IMRT) through Atlas registration.

MRI‐based treatment planning is a promising technique for liver SBRT, improving target volume delineation accuracy and reducing radiation dose to the OARs. However, MRI geometric distortion is a known important concern in MRI‐based treatment planning^17^, ^18^ and may cause dosimetric uncertainties, which is especially critical for liver SBRT due to its hypofractionation. It is therefore the goal of this study to quantitatively evaluate the dosimetric uncertainties caused by MR geometric distortion in MRI‐based liver SBRT treatment planning. We performed computer simulation studies based on measured MRI distortion data and clinical liver SBRT plans to evaluate the dosimetric effects of various scenarios of MRI distortion.

## 2 MATERIALS AND METHODS

### 2.A 3D MRI distortion simulation based on measured distortion data

3D MRI distortion was simulated based on sparsely measured MRI distortion data reported in the literature.^17^, ^18^ All MR images are expected to be distortion corrected using vendor's correction algorithms prior to any radiation therapy application. The simulated distortions consider only the system related distortions mainly caused by the inhomogeneities of main magnetic field B_0_ and nonlinearities of the gradient coils. And they selected a relatively high receiver bandwidth to acquire data to decreases the signal‐to‐noise ratio, while reducing the distortion caused by susceptibility and chemical shift. Therefore, the distortion measurement is mainly from the system related distortion caused by B_0_ inhomogeneities and gradient coil nonlinearities. Therefore, the simulated MRI distortion here refers to the residual MRI distortion, which is of concerned in MRI‐based treatment planning. Table**** 1 summarizes the sparsely measured residual MRI distortion data used in their study.^17^, ^18^ These data were measured on phantoms and were the averaged values of multiple investigated MRI sequences, including 2D Axial T1 FSE, 2D Axial T2 FRFSE, 3D CUBE T1, 3D CUBE T2, and 3D T1 3D SPGR. The in‐plane MRI distortions were calculated as the average of the distortion along the X and Y directions, and the through‐plane distortions were calculated along the Z direction.

**Table 1:** Residual MRI geometric distortions used in this study

Distance to center (mm)	100	150	200	250
In‐plan mean distortion (mm)	0.33	0.35	0.51	1.95
Through‐plan mean distortion (mm)	0.35	0.51	0.72	1.77

The simulated in‐plane and through‐plane MRI distortions, Dis_in_(*r*_1_) and Dis_thr_(*r*_2_) respectively, was obtained by fitting the sparsely measured in‐plane and through‐plane MRI distortions (Table 1) using a two‐term exponential fitting model as shown in Eq. (1a) and (1b),

(1a) $${\text{Dis}}_{\mathrm{in}}\left(r_{1}\right)=a_{1}\times e^{b_{1}\cdot r_{1}}+c_{1}\times e^{d_{1}\cdot r_{1}}$$

(1b) $${\text{Dis}}_{\mathrm{thr}}\left(r_{2}\right)=a_{2}\times e^{b_{2}\cdot r_{2}}+c_{2}\times e^{d_{2}\cdot r_{2}}$$

where *r*_1_ is the radial distance from each pixel at in‐plane image to the corresponding in‐plane image center (*x *=* *0, *y *=* *0) and *r*_2_ is the distance from each slice location to the central slice location (*z *=* *0). Then the radial distance *r* from each voxel to the scanning center (*x *=* *0, *y *=* *0, *z *=* *0) and the 3D MRI distortion Dis_sim_(*r*) was then calculated as Eq. (2a) and (2b):

(2a) $$r=r_{1}^{2}+r_{2}^{2}$$

(2b) $${\text{Dis}}_{\mathrm{sim}}\left(r\right)=\sqrt{{\text{Dis}}_{\mathrm{in}}{\left(r_{1}\right)}^{2}+{\text{Dis}}_{\mathrm{thr}}{\left(r_{2}\right)}^{2}}$$

Finally, a synthetic planning image dataset with simulated distortion was generated by deforming the original planning image dataset using the above‐determined 3D MRI distortion.

### 2.B 3D MRI distortion simulation based on body shrinkage

It was found from the above simulation study that meaningful anatomical changes due to MRI distortion mainly occur near the body surface.^17^, ^18^ To efficiently evaluate the dosimetric effects due to different distortion magnitudes, we used a simple “body shrinkage” method to generate the distorted planning images by shrinking the body contour with a preset value (2, 3, 4, and 5 mm) centripetally in the Eclipse™ Treatment Planning System.

### 2.C Dosimetric effects of MRI distortion in liver SBRT plan

Five clinical liver SBRT cases were included in this retrospective study. The planning CT images of the liver SBRT plans were used to generate simulated “distorted” CT planning images. For “distortion simulation” method, the simulated “distorted” CT planning images were generated by applying the MRI distortion (as a deformation field) onto the planning CT images via deformable image registration. For “body shrinkage” method, the simulated “distorted” CT planning images were generated by shrinking the body contour with a preset value (2, 3, 4, and 5 mm). For each liver SBRT case, we then generated two MRI‐based liver SBRT plans using the simulated “distorted” CT planning images:

Plan A, in which the dose is calculated on the simulated “distorted” planning CT images using the original CT numbers. Plan A is used to determine the dosimetric effects solely caused by MRI distortion, assuming that the MRI images can be precisely converted to CT images without errors in CT number assignment.

Plan B, in which the dose is calculated on the simulated “distorted” planning CT images with each organ/structure assigned with an organ‐specific CT number. This is to simulate a commonly‐used method of MRI‐to‐CT conversion via CT number assignment. In our study, assuming that the distortion to organs is relatively small, we just consider the distortion to the body surface. Therefore, in Plan B the organ‐specific CT number is assigned to structures of air, lungs and spine, which are contoured on the original CT images (without adding simulated distortion). The body structure needs to be recontoured after adding distortion and the soft tissue CT number is assigned to the new body structure. The organ‐specific CT numbers were determined as the average CT numbers of the organ/structure from five liver SBRT cases: −850 HU for air, −700 HU for the lungs, 225 HU for spine, and 0 HU for soft‐tissue. Plan B is used to determine the dosimetric effects caused jointly by MRI distortion and CT number assignment.

For both Plan A and Plan B, dose calculation was performed using the same plan parameters as in the original CT‐based liver SBRT plans. The prescription dose for four cases is 50 Gy and for the remaining one is 25 Gy. Dosimetric effects on various dose‐volume‐histogram (DVH) parameters were measured as the differences between the simulated MRI‐based plans (Plan A and Plan B) and the original CT‐based plans. The evaluated DVH parameters included: dose received at 95% of target volume (D_95_) for gross tumor volume (GTV) and planning target volume (PTV), dose received at 5% of target volume (D_5_) for GTV and PTV, maximum doses (*D*_max_) to spinal cord, *D*_max_ to stomach, *D*_max_ to small bowel, mean liver dose, the liver volume percentage receiving at least 30% of the prescription dose (*V*_30%_), and the volume percentage of PTV receiving at the prescription dose (*V*_Prescription_).

## 3 RESULTS

### 3.A Dosimetric effects of MRI distortion simulated using measured data

The fitted models of the in‐plane and through‐plane residual MRI distortions were determined as Eq. (3a) and (3b) respectively:

(3a) $${\text{Dis}}_{\mathrm{in}}\left(r_{1}\right)=0.0294\times e^{0.0164\cdot r_{1}}-0.0294\times e^{-10.1657\cdot r_{1}}$$

(3b) $${\text{Dis}}_{\mathrm{thr}}\left(r_{2}\right)=0.0680\times e^{0.0129\cdot r_{2}}-0.0680\times e^{-0.4647\cdot r_{2}}$$

**Figure 1:**
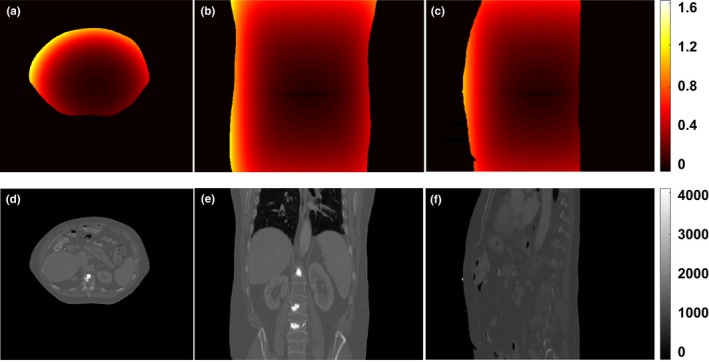
Simulated 3D MRI distortion map. (a–c) MRI distortion map at axial, coronal and sagittal view, (d–f) are corresponding original CT images.

**Figure 2:**
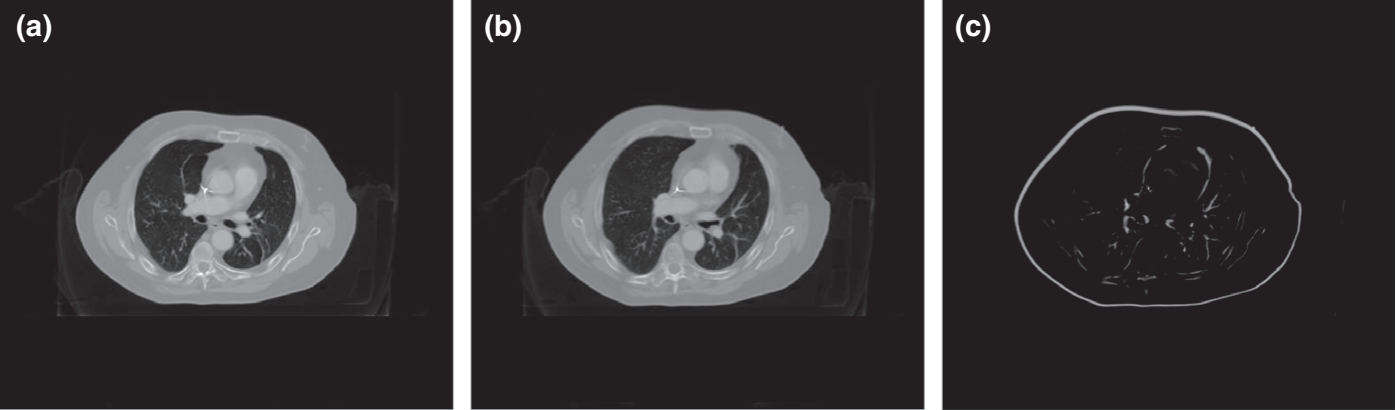
Simulated planning images with geometric distortion. (a) Original CT image of one slice position, (b) CT image of one slice position added with simulated distortion, and (c) intensity difference between original image and image with distortion.

Fig. 1 shows an example of the simulated 3D MRI distortion. It can be seen, as expected, that the magnitude of MRI distortion is minimal near the image center, and increases as *r* increases. The maximum magnitude of the simulated 3D MRI distortion ranged from 1.69 to 3.44 mm (mean: 2.77 mm) in the five liver SBRT cases. The average patient size (maximum diameter in the left to right direction) is 41.5 cm, with the maximum patient size is 45.7 cm and the minimum patient size is 38.8 cm. Fig. 2 shows an example of the planning CT images before and after applying the simulated 3D MRI distortion, and the intensity difference map between them. It can be seen that the anatomical difference caused by the MRI distortion mainly occurred near the body surface, resulting in the so‐called “body shrinkage” effect. The intensity differences within the body are minimal and have irregular patterns, implying that these differences are most likely averaged out by inter‐fractional patient positioning variations.

**Figure 3:**
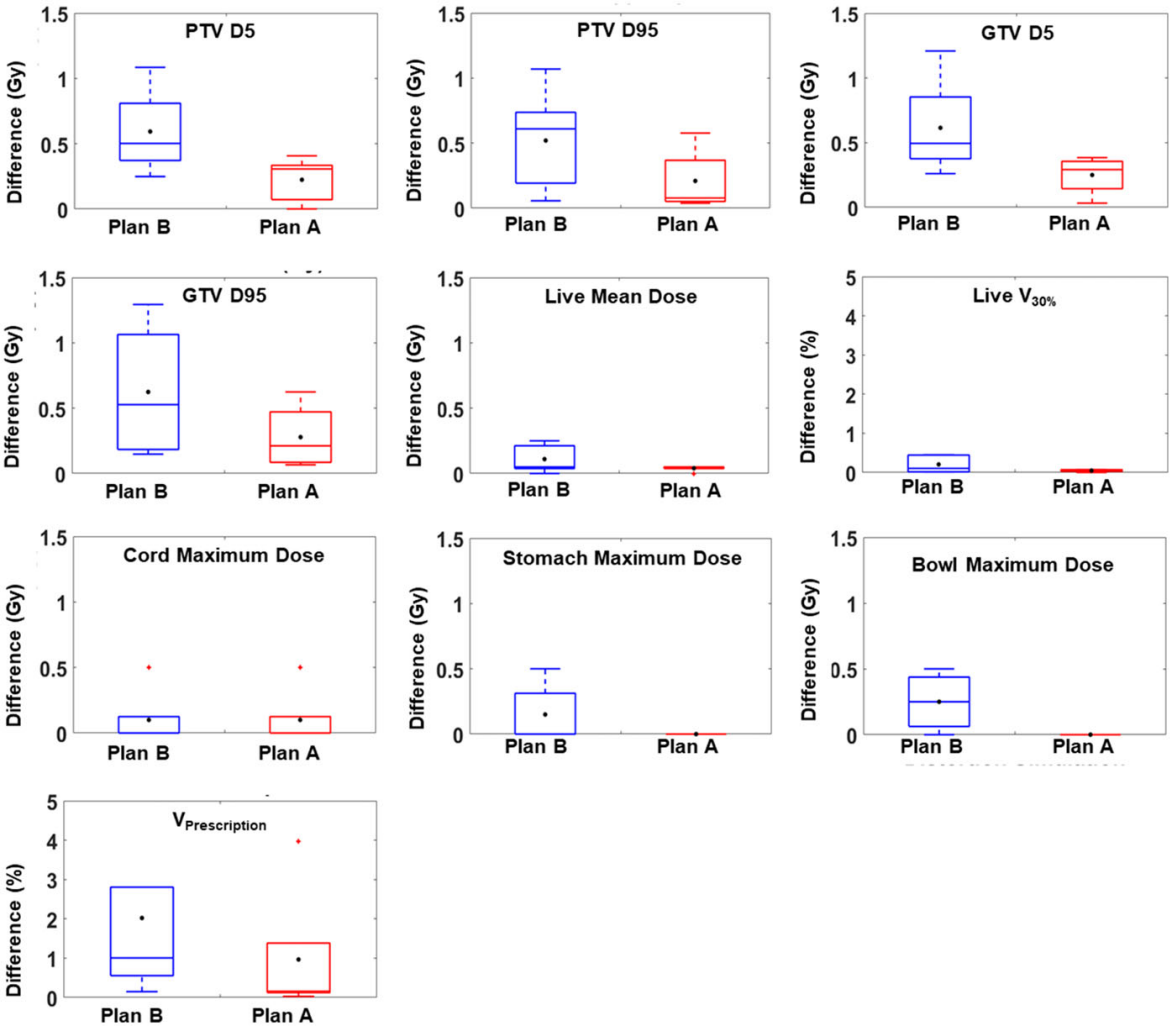
Dosimetric uncertainties caused by MR geometric distortions simulation on measured distortion data.

Fig. 3 shows the dosimetric effects on various DVH parameters caused by MRI distortions in two scenarios, Plan A (red boxes) and Plan B (blue boxes), respectively. In each box, the central line mark indicates the median and the central black dot indicates the average. The bottom and top edges of the box indicate the 25th and 75th percentiles, respectively. The whiskers are lines extending above and below each box. Whiskers are drawn from the ends of the interquartile ranges to the furthest observations within the whisker length (the adjacent values). Observations beyond the whisker length are marked as outliers. By default, an outlier is a value that is more than 1.5 times the interquartile range away from the top or bottom of the box. Outliers are indicated with a red + sign.

For Plan A, the differences in D_95_ and D_5_ for GTV and PTV averaged 0.24 ± 0.19 Gy. For the prescription dose of 50 Gy (including four cases), the maximum difference in D_95_ and D_5_ for GTV and PTV was 0.35 Gy and the maximum error percentage was 0.7%; for a prescription dose of 25 Gy, the maximum difference was 0.10 Gy and the maximum error percentage was 0.4%. And the differences in mean liver dose and liver *V*_30%_ were 0.04 Gy and 0.05% respectively. The differences in other DVH parameters were minimal (<0.1 Gy or <1.0%). For Plan B, the differences in D_95_ and D_5_ for GTV and PTV averaged 0.58 ± 0.38 Gy. For the prescription dose of 50 Gy (including four cases), the maximum average difference in D_95_ and D_5_ for GTV and PTV was 0.76 Gy and the maximum error percentage was 1.52%; for a prescription dose of 25 Gy, the maximum difference was 0.26 Gy and the maximum error percentage was 1.04%. And the differences in mean liver dose and liver *V*_30%_ were 0.11 Gy and 0.21%, respectively. The differences in other DVH parameters were also small (<0.1 Gy or <2.0%).

It can be observed from these results that: (a) dosimetric effects of MRI distortion on liver SBRT treatment plans are generally small, and (b) MRI‐to‐CT conversion using bulk CT number assignment tends to produce extra (but small) dosmetric uncertainties in addition to MRI distortion.

### 3.B Dosimetric effects of MRI distortion simulated using “body shrinkage” method

**Figure 4:**
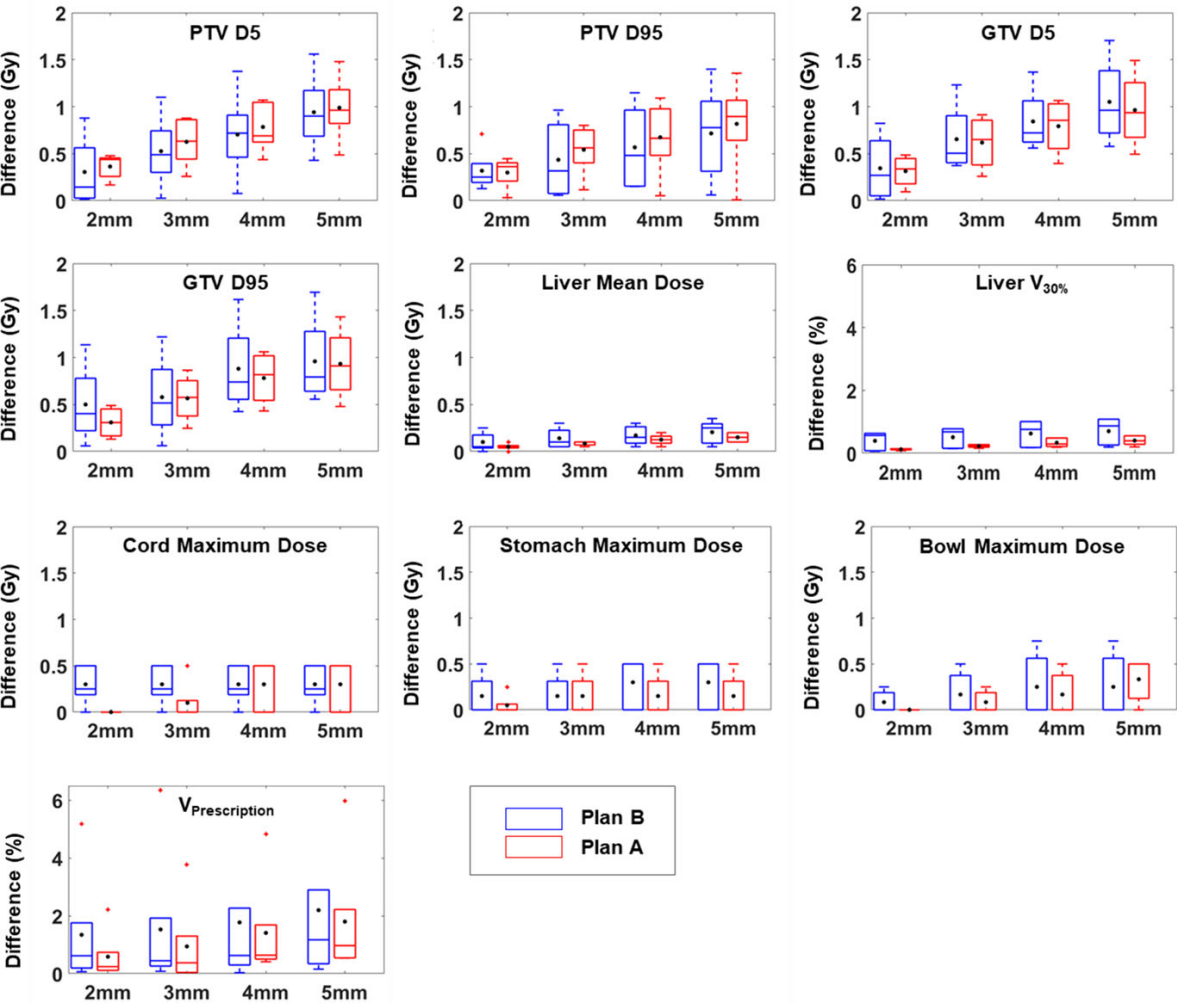
Dosimetric uncertainties caused by MR geometric distortions simulation based on “body shrinkage” method.

Fig. 4 shows the dosimetric effects on various DVH parameters caused by different MRI distortion magnitudes which are simulated using the “body shrinkage” method. It is generally observed that the dosimetric differences increase as the MRI distortion magnitude increases, despite that they are more prominent in some DVH parameters (such as PTV D_5_, PTV D_95_, liver V_30_%) than others (such as maximum dose to stomach).

The average maximal MRI distortion of the five liver SBRT cases is 2.77 mm as determined in our earlier simulation study. Using 2.77 mm as a referencing value for a typical maximal MRI distortion of a liver SBRT patient, we interpreted our results of the “body shrinkage” as follows. For Plan A, with the simulated centripetal MRI distortion (2–3 mm), the differences in D_95_ and D_5_ for GTV and PTV were generally small, with an average of 0.45 ± 0.24 Gy. For the prescription dose of 50 Gy (including four cases), the maximum difference in D_95_ and D_5_ for GTV and PTV was 0.74 Gy and the maximum error percentage was 1.48%; for a prescription dose of 25 Gy, the maximum difference was 0.26 Gy and the maximum error percentage was 1.04%. The differences in mean liver dose and liver V_30%_ ranged 0.05‐0.09 Gy and 0.12–0.23% respectively. The differences in other DVH parameters were small (<0.15 Gy or <1.0%). For Plan B, the average differences in D_95_ and D_5_ for GTV and PTV (with simulated MR distortion = 2‐3 mm) were still small, with an average of 0.45 ± 0.37 Gy. For the prescription dose of 50 Gy (including four cases), the maximum difference in D_95_ and D_5_ for GTV and PTV was 0.68 Gy and the maximum error percentage was 1.36%; for a prescription dose of 25 Gy, the maximum difference was 0.62 Gy and the maximum error percentage was 2.48%. The differences in mean liver dose and liver V_30%_ were 0.10–0.14 Gy and 0.39–0.51%, respectively. The differences in other DVH parameters were also small (<0.3 Gy or <1.6%).

## 4 DISCUSSION

Previous studies evaluated the geometric distortion and dosimetric effect using different approaches. Eleftherios et al.^19^ detected up to 2 mm geometric distortion for a range of radial distances up to approximately 135 mm by performing rigid spatial co‐registration between MR and CT images, and investigated the dosimetric uncertainties caused by spatial offsets of 0.5 up to 3 mm to the target locations. Christian et al.^20^ measured a displacement map by detecting the difference of markers in the phantom between MR and CT images, and evaluated the dosimetric uncertainties for prostate patient by applying the displacement map to CT images. Yue et al.^21^ fitted a second‐order polynomial model to map geometric distortions using measured 3D residual geometric distortion and evaluated the dosimetric uncertainties for gastrointestinal tract, genitourinary area, thoracic area, head and neck, and spine patients by applied the distortions to CT images.

In this study, we investigated the dosimetric effects caused by MRI geometric distortion in a computer simulation study. In addition, since the lack of electron density information in MRI is the other major impediments for MRI‐based treatment planning, we also considered the dosimetric effects caused by CT number assignment.

We tried to use a more realistic geometric distortion, but due to the chemical shift effect, different MR scanners and different vendor correction algorithm, we cannot verify the effect of all distortions on the MRI‐based treatment planning dose calculation. We simulated MR geometric distortion based on the measured distortion on a phantom scanned with a GE 1.5T MR‐SIM scanner. The distortion difference caused by different scanning machine and different patients cannot be evaluated and considered in this study.

For MR geometric distortion simulation from “body shrinkage” method, we consider the distortion at the surface of the body. It was found that the largest distortion occurs at the surface of the body, the organs inside the body still have distortion. If the tumor is closer to the center of the image, the distortion can be neglected. However, if the tumor is closer to the surface, due to the distortion, a larger margin may need to be considered for GTV contouring. For MR geometric distortion simulation based on measured distortion data, the distortion is added to both the body surface and the organs. However, in dose calculation, we did not recontour the organ structures since we wanted to compare the dose difference by minimizing the dose difference caused by the structure contouring uncertainties. In this approach, we did not consider the distortion of the organs in dose calculation.

Based on Figs 3 and 4, from Plan A to Plan B, the deviation in D_95_ and D_5_ (distortion simulation generated from measured distortion) increases by about 0.3 Gy, while the deviation in D_95_ and D_5_ (distortion simulation from “body shrinkage”) increases by only 0.02 Gy. The level of increase should have similar results for D_95_ and D_5_ (at 3 mm of distortion) for Plan A and Plan B. However, the increase is different. There are two reasons for the different level of increase. First of all, although 0.3 and 0.02 Gy are different, they are both very small values and may still be considered as “similar” considering the prescription dose is much higher. Secondly, for Fig. 4, the true distortion within the body is ignored. Depending on the location of the tumor, in the center or peripheral of the body, the dosimetric effect of such omission is different, which may have led to the differences as observed by the reviewer. And for Fig. 3, even though we did not recontour the organ structures after adding distortion, the CT number within the organ structures has been changed. These might cause the increase level different. This is a topic worth of further investigation.

In addition, for MR geometric distortion simulation based on the measured distortion data, we intentionally matched the imaging isocenter to the center of the MRI scanner. For the purpose of our study, it should be sufficient to quantify the magnitude of dosimetric impact of MR distortion to MRI‐based treatment planning. In cases of patient shifts in MRI scanner, the shifts are usually limited, and should not significantly change the results of our study. And since our results are averages of five patients, the effects of different patient shifts should already have been included (at least to some extent) in the results.

For liver SBRT, MRI‐based treatment planning presents a challenge of tumor motion. 4D imaging is an emerging technique to obtain comprehensive information of tumor motion. Owing to the superior soft tissue contrast and freedom from radiation dose, 4D‐MRI techniques have been recently developed to overcome the limitations of 4D‐CT in abdominal imaging.^22^, ^23^ Future studies need to be performed on 4D MRI‐based treatment planning for liver SBRT to investigate the dosimetric uncertainties caused by geometric distortion and CT number assignment.

## 5 CONCLUSION

We performed simulation study of dosimetric effects from MRI distortion on liver SBRT plans. It was found that dose uncertainties caused by MR distortion was generally small (<1 Gy), for various DVH parameters. These results may indicate that it is dosimetrically acceptable to perform MRI solely based treatment planning for liver SBRT despite uncertainties in residual MRI distortion and CT number assignment.

## CONFLICT OF INTEREST

There is no conflict of interest involved in this study.
